# 

**Published:** 1901-09-21

**Authors:** 


### NOTICE TO CORRESPONDENTS.

All MSS., Letters, Books for Review, and other matters intended for the Editor should be addressed The Editor, "The Hospital," The Hospital Building, 28 & 29 Southampton Street, Strand, W.C.

The Editor cannot undertake to return rejected MS., even when accompanied by stamped directed envelope.

**Notice to Advertisers and Subscribers.**

All Advertisements, Orders for Copies of The Hospital, and Business Communications should be addressed to **THE MANAGER (Not to the Editor),** The Hospital, 28 & 29 Southampton Street, Strand, London, W.C.

The Cost of Subscription, post free, is as follows:—Per week, 2½d.; per quarter, 2s. 8d.; per half-year, 5s. 3d.; per annum, 10s. 6d. Foreign:—Per annum, 15s. 2d., payable, in all cases, in advance.

**NOTICE.—Vol. XXX. of "The Hospital" (April 1901, to September 1901), in handsome cloth binding will shortly be on sale at the Office of this Journal, price, 7s. 6d. Cases for binding the Numbers contained in this Volume 1s. 6d. Index to Vol. XXX., 2½d., post free.**

**The Monthly Part for October will be ready on October 1st. Price 6d., by post 8d.**

### Scraps and Gleanings.

During the nineteenth century the European population has increased from 170,000,000 to 510,000,000.

Through the will of the late Mr. J. C. Ehemann the German Hospital at Dalston receives a legacy of £100.

The Aberdeen Hospital for Incurables is the recipient of a legacy of £100, under the will of the late Mr. W. Harvey.

As an innovation the Camberwell Guardians have decided to provide the boys under their control, numbering nearly 1,000, with nightshirts.

The new out-patients' department at the Bristol General Hospital is to cost upwards of £6,000 or £7,000; £3,000 of this has already been paid.

During the three years since the Hebburn Infirmary was opened 1,000 patients have been treated, of which number under 300 were in-patients.

The Registrar-General's return shows that the deaths registered last week in 33 great towns of England and Wales correspond to an annual rate of 19·4 of their aggregate population, estimated at 11,463,026 persons.

The number of attendances of district patients at the Dundee Royal Infirmary during the last quarter was 1,400, the attendances of out-patients was 9,352 as against 8,739 last year; and the in-patients numbered 758.

The legacies received by the Bristol General Hospital during the past year amounted to £5,302. The collections at places of worship showed an increase of £116. There had been a net decrease in expenditure of £274.

The first year's work of the Pasteur Institute in India is just completed. The total number of patients was 321, 146 of whom were Europeans and 175 natives. There was not a single failure in the treatment among Europeans, and only two amongst the natives.

The new accommodation in connection with the Belfast Hospital provides for ten nurses, but there are still 29 nurses left to sleep in the hospital building. It is considered advisable that there shall be a new home for nurses, and the Board of Guardians have accepted a tender for the erection of a nurses' home at a cost of £11,000, a portion of which accommodation it is thought should be set aside for the fever nurses.

### The Student of Medicine.

### Appointments.

**Wm. A. Anderson**, M.B., C.M.Edin., appointed Assistant Medical Officer to the Plymouth Borough Asylum, Ivybridge, Devon. **W. H. Andrews**, L.R.C.P., L.R.C.S.Edin., appointed District Medical Officer of the Kingsbridge Union. **R. H. Brew**, L.R.C.P., L.R.C.S.Edin., appointed Medical Officer of Health for the Clutton Rural District. **F. J. Butt**, M.B., C.M.Edin., appointed Medical Officer of Health for the Hoole Urban District. **Astley Vavasour Clarke**, M.D., B.C.Cantab., appointed Physician to the Leicester Infirmary and Fever House. **Alwyne T. Compton**, M.R.C.S., L.R.C.P., appointed Second Assistant Medical Officer, St. Mary Islington Infirmary, Highgate Hill, N. **T. B. Dakin**, M.R.C.S., L.R.C.P.Lond., appointed District Medical Officer of the Glanford Brigg Union. **T. E. Gordon**, M.D.Durh., appointed Medical Officer of Health for the Towyn Urban District. **J. Guest Gornall**, M.A., M.B., D.P.H.Cantab., appointed Medical Officer of Health for the County Borough of Warrington. **H. Hipwell**, M.R.C.S., L.R.C.P.Lond., appointed District Medical Officer of the Banbury Union. **T. H. Livingstone**, M.B., C.M.Edin., appointed District and Workhouse Medical Officer of the Weardale Union. **A. E. Peake**, M.R.C.S., L.R.C.P.Lond., appointed District and Workhouse Medical Officer of the Henley Union. **William H. Peile**, M.A.Camb., M.R.C.S., L.R.C.P., appointed First Assistant Medical Officer to the Bethnal Green Infirmary, Cambridge Heath, N.E. **F. W. Salter**, L.R.C.P., L.R.C.S.Edin., appointed District Medical Officer of the Newtown and Llanidloes Union. **Roland A. Stevenson**, L.R.C.P.Lond., M.R.C.S.Eng., appointed Junior Resident Medical Officer at the London Open-Air Sanatorium, Pinewood, Berkshire. **W. J. Thomas**, M.R.C.S., L.R.C.P.Lond., appointed District Medical Officer of the Merthyr Tydfil Union. **J. Troup**, M.B., C.M.Aberd., appointed District Medical Officer of the Bury Union. **Alexander Minter Watts**, M.B., B.S.Durh., M.R.C.S.Eng., L.R.C.P.Lond., appointed Medical Officer of Health to the Holbeach Urban District Council. **G. M. Y. Whittingham**, M.R.C.S., L.R.C.P.Lond., appointed Assistant Medical Officer to the Wandsworth and Clapham Union.

St. Thomas's Hospital.—The following gentlemen have been selected as House Officers from Tuesday, September 3rd., 1901:—House Physicians: **L. H. C. Birkbeck**, B.A., M.B., B.Ch.Oxon.; **V. S. Hodson**, B.A., M.B., B.Ch.Oxon. Assistant House Physicians: **W. M. Glanville**, B.A., M.B., B.Ch.Oxon.; **T. W. S. Paterson**, M.A., B.C.Cantab., L.R.C.P., M.R.C.S. House Surgeons: **C. A. R. Nitch**, M.B.Lond., L.R.C.P., M.R.C.S.; **W. H. O. Woods**, B.A., M.B., B.C.Cantab.; **S. Hunt**, L.R.C.P., M.R.C.S.; **A. S. Grimwade**, L.R.C.P., M.R.C.S. Assistant House Surgeons: **G. A. C. Shipman**, M.A., M.B., B.C.Cantab., L.R.C.P., M.R.C.S.; **W. Hill**, B.A. Cantab., L.R.C.P., M.R.C.S.; **T. W. H. Downes**, L.R.C.P., M.R.C.S.; **F. J. Child**, M.A., M.B., B.C.Cantab., L.R.C.P., M.R.C.S. Obstetric House Physicians (Senior): **H. S. Stannus**, L.R.C.P., M.R.C.S. (Junior): **Z. Mennell**, L.R.C.P. M.R.C.S. Ophthalmic House Surgeons (Senior): **H. S. Harris**, L.R.C.P., M.R.C.S. (Junior): **F. B. Skerritt**, B.Sc.Lond., L.R.C.P., M.R.C.S. Clinical Assistants in the Special Department for Diseases of the (Throat): **H. T. D. Acland**, L.R.C.P., M.R.C.S.; **A. D. Hamilton**, L.R.C.P., M.R.C.S.; (Skin): **F. Clarkson**, M.B. B.S.Durh.; **J. L. Timmins**, M.A., B.C.Cantab.; (Ear): **H. Spurrier**, B.A.Cantab., L.R.C.P., M.R.C.S. Clinical Assistant in the Electrical Department: **H. T. D. Ackland**, L.R.C.P., M.R.C.S.

